# The Changing Landscape of Antibiotic Treatment: Reevaluating Treatment Length in the Age of New Agents

**DOI:** 10.3390/antibiotics14070727

**Published:** 2025-07-20

**Authors:** Francesca Serapide, Salvatore Rotundo, Luca Gallelli, Caterina Palleria, Manuela Colosimo, Sara Palma Gullì, Gianmarco Marcianò, Alessandro Russo

**Affiliations:** 1 Department of Medical and Surgical Sciences, University ‘Magna Graecia’ of Catanzaro, 88100 Catanzaro, Italy; f.serapide@unicz.it (F.S.); a.russo@unicz.it (A.R.); 2 Infectious and Tropical Disease Unit, “Renato Dulbecco” Hospital, 88100 Catanzaro, Italy; srotundo91@gmail.com (S.R.); sarapalma08@gmail.com (S.P.G.); 3 Department of Health Science, University ‘Magna Graecia’ of Catanzaro, Viale Europa, 88100 Catanzaro, Italy; 4 Clinical Pharmacology and Pharmacovigilance Unit, “Renato Dulbecco” University Hospital Viale Europa, 88100 Catanzaro, Italy; palleria@unicz.it (C.P.); gianmarco.marciano3@gmail.com (G.M.); 5 Microbiology and Virology Unit, “Renato Dulbecco” University Hospital, 88100 Catanzaro, Italy; manuelacolosimo@hotmail.it

**Keywords:** antibiotics, pharmacokinetic, pharmacodynamic, half-life, resistance

## Abstract

Background: The landscape of antimicrobial therapy is undergoing a profound transformation; the contemporary arsenal of antimicrobials, particularly those with extended half-lives and enhanced tissue penetration, necessitates critically reassessing these traditional paradigms. The growing emphasis on antimicrobial stewardship programs has underscored the importance of optimizing antimicrobial agents to minimize the development and spread of resistance. Shorter treatment durations, when clinically appropriate, represent a key strategy in this endeavor. Methods: This narrative review provides a comprehensive synthesis of current evidence on the duration of antimicrobial therapy, with a particular focus on the clinical and pharmacological implications of novel agents, including long-acting formulations. Results: We critically examine the pharmacokinetic and pharmacodynamic properties of these agents, evaluate the opportunities and limitations associated with treatment shortening strategies, and underscore the pivotal role of antimicrobial stewardship in optimizing therapeutic outcomes within an increasingly complex and evolving landscape. Conclusions: The future of antimicrobial therapy lies in a personalized approach, where treatment decisions are tailored to the individual patient, but detailed clinical trials are necessary to evaluate these approaches.

## 1. Introduction

The landscape of antimicrobial therapy is undergoing a profound transformation, driven by the emergence of novel agents possessing distinct pharmacokinetic and pharmacodynamic characteristics [1,2,3,4,5]. For decades, the duration of antimicrobial treatment has been largely guided by empirical practices and historical precedents, often rooted in clinical trials conducted with older drug classes [6,7]. However, the contemporary arsenal of antimicrobials, particularly those with extended half-lives and enhanced tissue penetration, necessitates critically reassessing these traditional paradigms [8].

Conventional antimicrobial therapy has been based on the idea of “sufficient duration” to kill the infection and stop recurrence [9]. While effective in many cases, this approach has often resulted in prolonged treatment courses, exposing patients to unnecessary side effects [10,11], increasing healthcare costs, and contributing to the escalating problem of antimicrobial resistance [4]. The advent of new antimicrobials, e.g., lipoglycopeptides [8,12,13,14], novel cephalosporins [15,16,17], and long-acting aminoglycosides, challenges this established dogma by offering the potential for shorter treatment durations without sacrificing clinical efficacy [18,19,20,21].

These novel agents often exhibit distinct pharmacokinetic and pharmacodynamic profiles compared to their predecessors. For instance, long-acting lipoglycopeptides provide sustained drug concentrations over extended periods, supporting longer intervals between doses and abbreviating the treatment regimen [22,23]. Similarly, certain novel cephalosporins demonstrate enhanced activity against multidrug-resistant organisms and achieve high tissue concentrations, suggesting the possibility of truncating the treatment course in specific infections [15,16,17,24].

Furthermore, the growing emphasis on antimicrobial stewardship programs has underscored the importance of optimizing antimicrobial agents to minimize the development and spread of resistance [25]. Shorter treatment durations, when clinically appropriate, represents a key strategy in this endeavour. By reducing the overall exposure to antimicrobials, we could theoretically mitigate the selective pressure that promotes resistance and preserve the effectiveness of existing drugs [26,27].

In addition to clinical trials, the development and validation of rapid diagnostic tests are crucial for identifying patients who may benefit from shorter treatment courses [28]. These tests can provide timely information on pathogen identification, susceptibility, and treatment response, enabling clinicians to make informed decisions about antimicrobial therapy duration [28].

This narrative review aims to synthesize the current evidence regarding the duration of antimicrobial therapy, with a focus on the implications of novel agents, including long-acting formulations. We will explore the pharmacokinetic and pharmacodynamic characteristics of these drugs, discuss the challenges and opportunities associated with shortening treatment durations, and highlight the importance of antimicrobial stewardship in this evolving landscape.

## 2. Pharmacokinetic and Pharmacodynamic Considerations

The rational design and optimization of antimicrobial therapy hinge upon a deep understanding of pharmacokinetic (PK) and pharmacodynamic (PD) principles [29]. These two domains, intricately intertwined, dictate the drug’s journey through the body and its interaction with the target pathogen, respectively [30]. In the era of novel antimicrobials, particularly long-acting agents, a nuanced appreciation of these concepts is paramount for redefining treatment durations [31].

Traditionally, antimicrobial dosing strategies have often been predicated on achieving peak concentrations or maintaining drug levels above a certain threshold for a predefined duration [32]. However, the emergence of agents with extended half-lives, unique distribution patterns, and novel mechanisms of action necessitates a paradigm shift towards a more sophisticated approach.

In Table 1, we summarized the pharmacokinetic and pharmacodynamic characteristics of new antimicrobials.

Long-acting lipoglycopeptides, such as dalbavancin and oritavancin, exemplify this shift [22,23]. These agents exhibit prolonged terminal elimination half-lives, often exceeding several days, allowing for infrequent dosing and potentially abbreviated treatment courses [33]. This pharmacokinetic profile translates into sustained drug exposure, which, in turn, profoundly influences the pharmacodynamic parameter of time above the minimum inhibitory concentration (T > MIC) [34].

Table 2 lists the following pharmacodynamic markers to enhance antimicrobial treatment.

The T > MIC, a critical determinant of antimicrobial efficacy for time-dependent antibiotics, reflects the duration for which the drug concentration surpasses the pathogen’s MIC [35]. For long-acting agents, even with gradually declining concentrations over time, the extended half-life ensures that the T > MIC remains adequate for serial eradication [36]. This concept challenges the conventional notion that higher peak concentrations are always superior, highlighting the importance of sustained drug exposure in achieving optimal outcomes.

Furthermore, the area [29,37,38] under the concentration-time curve (AUC) to MIC ratio (AUC/MIC) is another pivotal pharmacodynamic index, particularly for concentration-dependent antibiotics such as aminoglycosides and fluoroquinolones. The AUC/MIC reflects the overall drug exposure relative to the pathogen’s susceptibility [39]. Novel aminoglycoside formulations, such as liposomal amikacin, demonstrate enhanced intracellular penetration and prolonged drug release, resulting in higher AUC/MIC values and potentially allowing for shorter treatment durations [40].

Beyond these traditional PK/PD parameters, the concept of post-antibiotic effect (PAE) plays a crucial role in optimizing antimicrobial therapy [41]. The PAE refers to the persistent suppression of bacterial growth after a brief exposure to an antibiotic [42]. Certain novel agents exhibit prolonged PAEs, extending beyond the period of detectable drug concentrations [43]. This phenomenon allows for less frequent dosing and potentially shorter treatment courses, as the continued suppression of bacterial growth contributes to overall efficacy [44].

Moreover, the understanding of drug penetration into various tissue compartments is essential for optimizing antimicrobial therapy [45]. Novel agents often exhibit enhanced tissue penetration, allowing for effective treatment of infections in difficult-to-reach sites [46]. For example, certain cephalosporins demonstrate excellent penetration into the cerebrospinal fluid, making them suitable for the treatment of meningitis [46].

The antibiotic protein-binding property plays a pivotal role [47]. Antibiotics that are highly protein-bound can have a lower free concentration of the active portion [48]. Sometimes, despite the high concentration of the total drug, its free form can be low, with an impact on the antibiotic effectiveness [49]. This is important, especially in the setting of hypoalbuminemia, where the free drug concentration can be higher than expected [50].

Furthermore, the concept of adaptive resistance must be considered [51]. Some bacteria can adapt to the presence of antibiotics, leading to a temporary increase in their MIC [52]. This phenomenon can impact the effectiveness of time-dependent antibiotics, as the T > MIC may be reduced [53]. Understanding the mechanisms of adaptive resistance is crucial for optimizing dosing regimens and preventing treatment failure.

In addition, the microbiome, the community of microorganisms that reside in the human body, can also be affected by antibiotic therapy [54]. Broad-spectrum antibiotics can disrupt the balance of the microbiome, leading to adverse effects such as Clostridioides difficile infection [55]. Understanding the impact of antibiotics on the microbiome is essential for minimizing these adverse effects and promoting patient health [56].

**Table 1:** The Pharmacokinetic and Pharmacodynamic Characteristics of New Antimicrobials.

Antimicrobial Class	Example Agents	Key PK/PD Characteristics	Potential Impact on Therapy Duration	Additional Notes
Lipoglycopeptides [57]	Dalbavancin, Oritavancin	Long half-life (>7 days), sustained drug exposure, high tissue penetration	Enables single-dose or infrequent dosing, reducing treatment duration	Useful in outpatient settings (e.g., OPAT)
Novel Cephalosporins [58]	Ceftolozane-Tazobactam, Ceftazidime-Avibactam, Cefiderocol, Cefepime-enmetazobactam, Ceftobiprole	Enhanced activity against MDR organisms, high tissue concentrations, stability against beta-lactamases	May allow shorter therapy durations for MDR infections, particularly in pneumonia and complicated UTI	Cefiderocol has activity against carbapenem-resistant Gram-negative bacteria
Long-Acting Aminoglycosides [59]	Liposomal Amikacin, Plazomicin	Improved intracellular penetration, prolonged drug release, and concentration-dependent killing	Higher AUC/MIC ratios enable reduced dosing frequency	Suitable for nosocomial pneumonia and ventilator-associated pneumonia
Beta-Lactam/Beta-Lactamase Inhibitors [60,61]	Meropenem-Vaborbactam, Imipenem-Relebactam	Broad-spectrum activity, effective against carbapenem-resistant pathogens	Potential to shorten therapy for multidrug-resistant infections	Enhanced stability against serine beta-lactamases
Fluoroquinolones [62]	Delafloxacin	Dual activity against Gram-positive and Gram-negative bacteria, high intracellular penetration	Potentially shorter therapy in pneumonia and SSTIs	Lower risk of resistance development compared to other fluoroquinolones

**Table 2:** Pharmacodynamic Markers to Enhance Antimicrobial Treatment.

Pharmacodynamic Index	Definition	Importance of Novel Agents	Clinical Implications
T > MIC (Time above MIC)	Duration drug concentration remains above MIC	Essential for time-dependent antibiotics (e.g., beta-lactams, lipoglycopeptides)	Higher values correlate with improved bacterial eradication
AUC/MIC (Area under the Curve to MIC Ratio)	Total drug exposure relative to MIC	Critical for concentration-dependent antibiotics (e.g., aminoglycosides, fluoroquinolones)	Optimizing this ratio allows for extended dosing intervals
Post-Antibiotic Effect (PAE)	Suppression of bacterial growth post-exposure	Longer PAE allows for extended dosing intervals and shorter courses	Important for aminoglycosides and fluoroquinolones, reducing toxicity risks
Cmax/MIC (Peak Concentration to MIC Ratio)	The ratio of maximum serum concentration to MIC	The key for concentration-dependent antibiotics (e.g., aminoglycosides)	Higher peaks enhance bacterial killing and reduce resistance development

In summary, the pharmacokinetics and pharmacodynamics of novel antimicrobials, particularly long-acting agents, offer unique opportunities to redefine antimicrobial therapy duration [29,30,34]. Taking into account these principles, clinicians can optimize dosing regimens, shorten treatment courses, and minimize the risk of adverse events and resistance development. Continued research and collaboration are essential to ensure that these advancements are translated into clinical practice.

## 3. Key Points

### 3.1. Novel Antimicrobials

- The pharmacokinetics and pharmacodynamics of new antimicrobials often differ significantly from traditional agents. For example, some exhibit prolonged post-antibiotic effects, allowing for less frequent dosing and potentially shorter treatment durations.
- Long-acting formulations, such as lipoglycopeptides and long-acting liposomal aminoglycosides, provide sustained drug concentrations, potentially enabling shorter treatment courses for certain infections.

### 3.2. Pharmacodynamic Indices

- Optimizing antimicrobial therapy requires a thorough understanding of pharmacodynamic indices, such as the ratio of the area under the concentration-time curve to the minimum inhibitory concentration (AUC/MIC) and the time above the MIC.
- These indices can guide the selection of appropriate dosing regimens and inform decisions regarding treatment duration.

### 3.3. Clinical Implications

The translation of pharmacokinetic and pharmacodynamic principles into clinical practice redefines antimicrobial therapy durations. The emergence of novel antimicrobials, particularly those with long-acting properties, requires a paradigm shift moving towards more personalized and efficient strategies [63].

One of the most significant clinical implications lies in the possibility to shorten treatment courses [64] (Table 3).

For numerous infectious diseases, traditional durations have been established based on historical precedents, often without robust evidence supporting prolonged therapy [64]. With newer agents, especially those exhibiting sustained drug exposure, the possibility of abbreviating treatment becomes a tangible reality [78,79]. This is particularly relevant in infections where clinical response is rapid and sustained, such as uncomplicated skin and soft tissue infections or urinary tract infections [79].

The concept of outpatient parenteral antimicrobial therapy (OPAT) is also revolutionized by long-acting antimicrobials [80,81]. Traditionally, OPAT required frequent infusions, often necessitating daily visits to healthcare facilities or the placement of indwelling catheters [82]. However, agents like dalbavancin or oritavancin, with their extended half-lives, allow for single-dose or infrequent dosing regimens, significantly simplifying OPAT and enhancing patient convenience [83]. This approach not only reduces healthcare costs but also improves patients’ quality of life, minimizing the impact on daily habits [84].

Additionally, there is a significant impact on antimicrobial stewardship initiatives. Reducing treatment times is a natural fit with stewardship’s fundamental goals of maximizing the use of antibiotics and reducing the emergence of resistance [85]. Reducing the overall exposure to antibiotics, it is potentially possible to mitigate the selective pressure that drives resistance, preserving the effectiveness of these critical drugs [9,63]. This strategy is especially crucial given the growing prevalence of multidrug-resistant organisms.

The clinical implications extend to specific patient populations as well. For example, immunocompromised patients, often at higher risk of severe infections, may benefit from the sustained drug exposure provided by long-acting agents [86]. Less frequent doses can increase adherence and lessen the burden of medication for these patients, who may need therapy for an extended length of time [87].

Several factors need to be carefully considered before shortened treatment courses are used [64]: both the type and severity of infection; the type of pathogen involved; and the clinical characteristics of the patient [88]. Clinical trials are essential to establish evidence-based guidelines for specific infections and patient populations.

Some biomarkers, such as procalcitonin, can be used to distinguish between bacterial and viral illnesses and track treatment response; therefore, their role in tailoring therapy is particularly significant [89,90,91].

The concept of de-escalation is also a key component of antimicrobial stewardship [92]. This involves transitioning from broad-spectrum antibiotics to narrower-spectrum agents once the pathogen is identified and susceptibility testing is available [92]. Shortening the duration of broad-spectrum therapy can help to minimize the risk of resistance and adverse effects [93].

In summary, the clinical implications of redefining antimicrobial therapy durations are far-reaching. The emergence of novel agents, particularly long-acting antimicrobials, offers the potential for shortened treatment courses, simplified OPAT, and enhanced antimicrobial stewardship. However, careful consideration of patient-specific factors, robust clinical trials, and the use of biomarkers are essential to ensure the safe and effective implementation of these strategies.

## 4. Key Points

### 4.1. Infection-Specific Considerations

- The optimal duration of antimicrobial therapy differs according to the type and severity of infection, the pathogen involved, and the patient’s underlying health status.
- For certain infections, such as uncomplicated urinary tract infections and some skin and soft tissue infections, shorter treatment courses are non-inferior to longer courses.

### 4.2. Antimicrobial Stewardship

- Shortening antimicrobial therapy duration is a key component of antimicrobial stewardship programs, which aim to optimize antimicrobial use and minimize the development of resistance.
- De-escalation strategies, such as transitioning from intravenous to oral therapy and shortening treatment duration based on clinical response, can help reduce unnecessary antimicrobial exposure.

### 4.3. Long-Acting Antimicrobials

- The use of long-acting antimicrobials may allow for outpatient parenteral antimicrobial therapy (OPAT) in patients who would otherwise require prolonged hospitalization.
- This approach can improve patient quality of life, reduce healthcare costs, and minimize the risk of hospital-acquired infections.

## 5. Challenges and Future Directions

One of the foremost challenges lies in the need for robust clinical trial data. Traditionally, antimicrobial therapy durations have been established based on trials designed with older drug classes and often with endpoints focused on clinical cure at the end of therapy [94]. However, with the emergence of long-acting agents and the emphasis on shorter treatment courses, new clinical trials evaluating pharmacokinetic and pharmacodynamic endpoints, as well as long-term clinical outcomes, are necessary to provide a comprehensive assessment of efficacy and safety.

Furthermore, the heterogeneity of patient populations poses a significant challenge. Factors such as age, comorbidities, and renal or hepatic impairment can significantly impact drug pharmacokinetics and pharmacodynamics, necessitating individualized treatment approaches [95,96]. This requires a shift to personalized medicine, where treatment decisions are tailored to the specific patient [97].

The development and validation of rapid diagnostic tests are crucial for guiding antimicrobial therapy. These tests can provide timely information on pathogen identification, susceptibility, and treatment response, enabling clinicians to make informed decisions about treatment duration [98,99,100]. For example, biomarkers such as procalcitonin can help to differentiate between bacterial and viral infections, as well as monitor the response to treatment [89].

The emergence of antimicrobial resistance remains a significant concern [101]. Shortening treatment durations, while generally beneficial, could potentially select resistant strains if not implemented judiciously [102]. Therefore, ongoing surveillance and monitoring of resistance patterns are essential to ensure that these strategies do not contribute to the spread of resistance.

The role of the microbiome in human health is increasingly recognized [103]. Broad-spectrum antibiotics can disrupt the delicate balance of the microbiome, leading to adverse effects such as *Clostridioides difficile* infection [104,105]. Future research should focus on developing strategies to minimize the impact of antibiotics on the microbiome, such as the use of narrow-spectrum agents or adjunctive therapies like probiotics or fecal microbiota transplantation [104].

The development of new antimicrobial agents is also crucial [106]. Despite the progress made in recent years, the pipeline of novel antibiotics remains limited [107]. Efforts to incentivize research and development in this area are essential to combat the growing threat of antimicrobial resistance [108].

The integration of artificial intelligence (AI) and machine learning (ML) into antimicrobial stewardship programs holds great promise [109]. AI and ML algorithms can analyze large datasets of clinical, microbiological, and pharmacokinetic data to identify patterns and predict optimal treatment durations [110,111].

The concept of therapeutic drug monitoring (TDM) is also gaining traction [112]. TDM involves measuring drug concentrations in patients’ blood or other body fluids to ensure that they are within the therapeutic range [113]. This approach can be particularly useful for optimizing dosing regimens of antibiotics with narrow therapeutic indices [114,115].

The role of combination therapy is also being explored [116]. Combining two or more antibiotics with different mechanisms of action can potentially broaden the spectrum of activity, enhance efficacy, and delay the emergence of resistance [21].

In summary, the challenges and future directions in redefining antimicrobial therapy durations are multifaceted. Robust clinical trials, personalized medicine, rapid diagnostics, antimicrobial stewardship, microbiome research, new drug development, AI/ML, TDM, and combination therapy are all crucial components of this evolving landscape.

## 6. Key Points

### 6.1. Clinical Trials

- Well-designed clinical trials are needed to evaluate the safety and efficacy of shortened antimicrobial therapy durations with novel agents.
- These trials should incorporate pharmacokinetic and pharmacodynamic data, as well as clinical outcomes, to inform optimal treatment strategies.

### 6.2. Personalized Medicine

- The future of antimicrobial therapy lies in personalized medicine, where treatment decisions are tailored to the individual patient.
- This approach requires the integration of clinical, microbiological, and pharmacokinetic data to optimize treatment duration and minimize the risk of adverse events.

### 6.3. Antimicrobial Resistance

- Careful monitoring of antimicrobial resistance patterns is crucial to ensure that shortened treatment courses do not contribute to the emergence of resistance.
- Antimicrobial stewardship programs play a vital role in preventing the spread of resistance.

Table 4 summarizes the possible strategies for Antimicrobial Stewardship to maximize therapy length.

### 6.4. Leveraging Artificial Intelligence to Optimize and Abbreviate Antimicrobial Therapy

The era of data-driven medicine has ushered in unprecedented opportunities to refine clinical decision-making, particularly in the realm of infectious disease management [117]. Artificial Intelligence (AI), with its capacity to process vast amounts of complex data, holds the huge promise for optimizing and potentially abbreviating antimicrobial therapy durations [118].

Traditional approaches to determining antibiotic treatment length have often relied on empirical guidelines and clinical experience, which may not always account for the intricate interplay of patient-specific factors, pathogen characteristics, and drug pharmacokinetics [29]. AI offers the potential to transcend these limitations by integrating diverse data streams to generate personalized treatment recommendations [119].

One of the most promising applications of AI lies in its ability to analyse electronic health records (EHRs) to identify patterns and predict treatment outcomes. EHRs contain a wealth of information, including patient demographics, laboratory results, imaging findings, and medication histories [120]. AI algorithms can sift through this data to identify patients who are likely to respond rapidly to treatment and may benefit from shorter durations [121].

Machine learning (ML) algorithms, a subset of AI, can be trained on large datasets of clinical data to develop predictive models for treatment response [122]. For instance, ML models can be trained to predict the probability of treatment success based on patient characteristics, pathogen virulence factors, and drug PK/PD parameters [122,123,124]. These models can then be used to identify patients who are at low risk of treatment failure and may be candidates for abbreviated therapy.

AI can also play a crucial role in optimizing antimicrobial dosing [125]. By integrating PK/PD data with patient-specific factors, AI algorithms can calculate personalized dosing regimens that maximize drug exposure at the infection site while minimizing the risk of toxicity [126]. This approach can be particularly valuable for antibiotics with narrow therapeutic indices or for patients with altered pharmacokinetics, such as those with renal or hepatic impairment [45].

Furthermore, AI can facilitate the interpretation of complex microbiological data [127]. The emergence of rapid molecular diagnostics and next-generation sequencing has generated a vast amount of data on pathogen identification and resistance mechanisms [128]. AI algorithms can analyse this data to provide clinicians with real-time insights into pathogen susceptibility and resistance patterns, enabling them to select the most appropriate antibiotic and optimize treatment duration [109,129].

AI can also be used to monitor treatment response in real time [130]. By analysing continuous streams of data from wearable sensors and other monitoring devices, AI algorithms can detect early signs of treatment failure or adverse events, allowing for prompt intervention [131]. This approach can be particularly valuable for patients receiving outpatient parenteral antimicrobial therapy (OPAT), who may be at risk of complications [132].

The integration of AI into antimicrobial stewardship programs holds great promise for optimizing antibiotic use and minimizing the development of resistance [133]. AI algorithms can analyse prescribing patterns to identify opportunities for de-escalation, dose optimization, and duration reduction [134]. This approach can help to ensure that antibiotics are used judiciously and that treatment durations are tailored to the individual patient’s needs.

However, the implementation of AI in antimicrobial therapy carries many challenges. One of the foremost challenges is the need for high-quality data [125]. AI algorithms are only as good as the data they are trained on. Therefore, it is essential to ensure that EHRs and other data sources are accurate, complete, and standardized [135].

Another challenge is the need for validation. AI algorithms must be rigorously validated in clinical trials before they can be widely implemented [136]. This process can be time-consuming and expensive, but it is essential to ensure that AI-driven treatment recommendations are safe and effective [137].

Despite these challenges, the potential benefits of AI in optimizing and abbreviating antimicrobial therapy are substantial. By integrating diverse data streams and leveraging advanced analytics, AI can help personalize treatment, improve outcomes, and minimize the risk of resistance.

## 7. Key Points

### 7.1. Data-Driven Personalization

- AI enables personalized treatment by analyzing vast EHR data and tailoring antibiotic durations to individual patient profiles.
- Machine learning algorithms predict treatment response, identifying patients suitable for shorter antibiotic courses.
- AI refines antibiotic dosing through PK/PD analysis, ensuring optimal drug exposure and minimizing toxicity.
- AI aids in interpreting complex microbiological data, facilitating rapid pathogen identification and resistance detection.

### 7.2. Real-Time Monitoring

- AI monitors treatment response via wearable sensors and devices, enabling timely intervention and preventing complications.
- AI optimizes antibiotic use in stewardship programs, promoting de-escalation and duration reduction.

### 7.3. Data Quality and Validation

- High-quality data and rigorous clinical trial validation are essential for safe and effective AI implementation.
- The use of AI requires the integration of many medical data sources and the use of a multidisciplinary team.
- AI is the future of medicine, and its use will increase, especially in the field of infectious disease.

**Figure 1:**
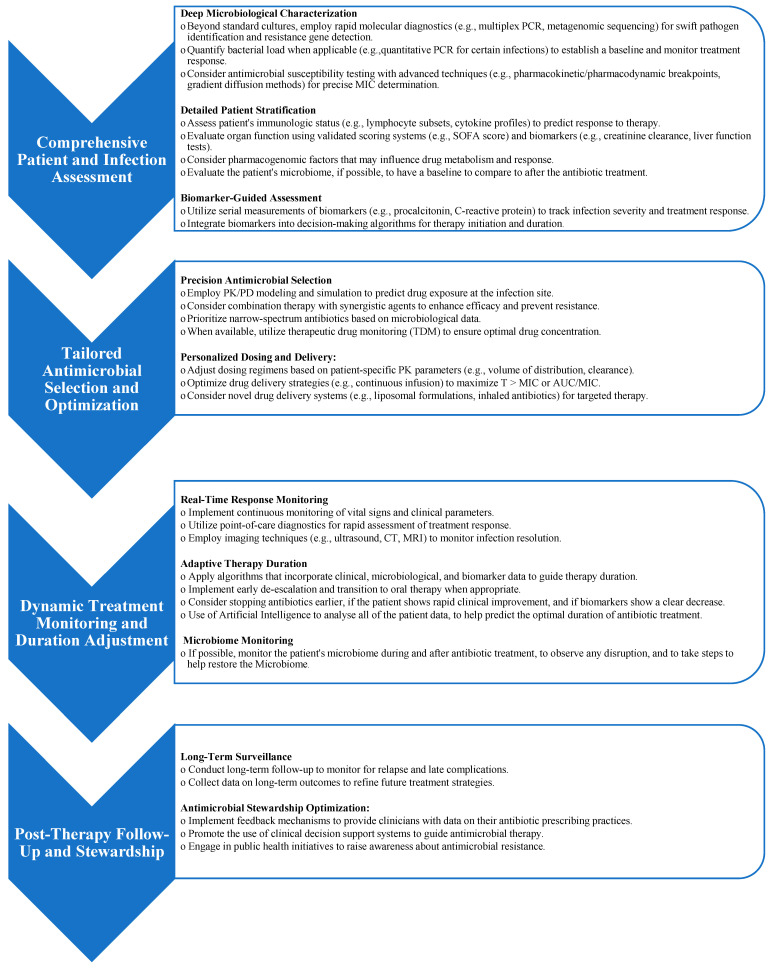
Advanced Diagnostic-Therapeutic Algorithm for Personalized Antimicrobial Therapy Duration.

Figure 1 shows the algorithm for personalized antimicrobial therapy duration following a targeted strategy, in relation to its main features.

**Figure 2:**
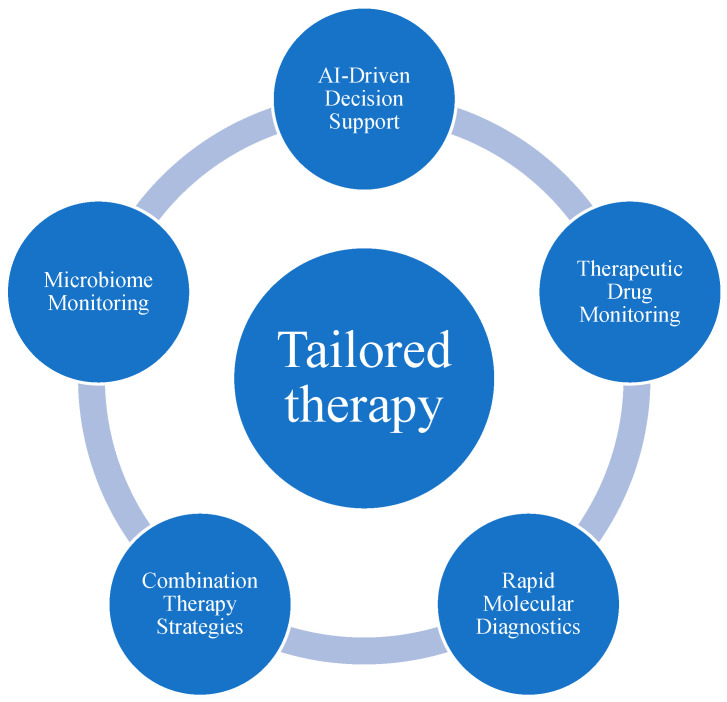
Future Directions in Personalized Antimicrobial Therapy.

Figure 2 summarizes possible future directions in personalized antibiotic therapy.

## 8. Conclusions

The development of new agents, the rise in antimicrobial resistance, and the increased focus on antimicrobial stewardship are all contributing factors to the ongoing change in the field of antimicrobial therapy. The distinct pharmacokinetic and pharmacodynamic characteristics of novel medications, especially long-acting medicines, are challenging the conventional paradigms of antimicrobial therapy duration. There are many advantages to the possibility of shorter treatment durations, such as lower medical expenses, more convenient patient care, and a lower chance of side effects. A strong scientific basis is necessary for the shift to shorter durations, though, and this includes the creation of quick diagnostic tools, pharmacokinetic and pharmacodynamic research, and well-planned clinical trials. Programs for antimicrobial stewardship are essential for maximizing the use of antibiotics and reducing the emergence and spread of resistance. Programs for antimicrobial stewardship are essential for maximizing the use of antibiotics and reducing the emergence and spread of resistance. One important tactic in this effort is to shorten treatment durations when clinically acceptable. We may be able to lessen the selection pressure that leads to resistance and maintain the efficacy of currently available medications by lowering the total exposure to antimicrobials. Antimicrobial stewardship programs that incorporate AI and machine learning have a lot of potential for optimizing therapy duration and tailoring treatment choices. Large clinical, microbiological, and pharmacokinetic data sets can be analyzed by AI algorithms to find trends and forecast the best possible treatment results. The future of antimicrobial therapy lies in a personalized approach, where treatment decisions are tailored to the individual patient, considering factors such as the type and severity of infection, the pathogen involved, the patient’s underlying health status, and pharmacokinetic and pharmacodynamic considerations. This approach requires a multidisciplinary effort, involving clinicians, microbiologists, pharmacists, and data scientists, working together to ensure that antimicrobial therapy is both effective and safe. Continued research and innovation are essential to address the challenges of antimicrobial resistance and optimize the use of these life-saving drugs. Antimicrobial therapy can continue to be beneficial for future generations if we embrace new technologies, encourage teamwork, and follow antimicrobial stewardship guidelines.

However, there needs to be a strong scientific basis for the shift to shorter antimicrobial therapy durations. Establishing the best treatment plans for these innovative drugs requires well planned clinical studies that consider pharmacokinetic and pharmacodynamic endpoints. To optimize clinical results and customize medication, these trials should also take patient-specific variables like age, comorbidities, and renal function into account.

## Figures and Tables

**Table 3:** Clinical Applications of Shortened Antimicrobial Therapy.

Infection Type	Traditional Duration	Evidence-Based Shortened Duration	Supporting Data
Uncomplicated UTI	7–10 days	3–5 days	Clinical trials suggest non-inferiority [65,66]
Skin and Soft Tissue Infections	10–14 days	5–7 days	Rapid clinical response allows a shorter duration [67,68,69,70,71]
Gram-Negative Bacteremia	14 days	7 days	Studies show equivalent efficacy [72,73,74,75]
Hospital-Acquired Pneumonia (HAP)	10–14 days	7–8 days	Shorter courses reduce resistance [11,76]
Intra-Abdominal Infections	7–10 days	4–6 days	De-escalation strategies allow early discontinuation [11,12,13,14,15,16,17,18,19,20,21,22,23,24,25,26,27,28,29,30,31,32,33,34,35,36,37,38,39,40,41,42,43,44,45,46,47,48,49,50,51,52,53,54,55,56,57,58,59,60,61,62,63,64,65,66,67,68,69,70,71,72,73,74,75,76,77]

**Table 4:** Strategies for Antimicrobial Stewardship to Maximize Therapy Length.

Strategy	Implementation	Expected Benefits	Key Considerations
De-escalation	The transition from broad to narrow-spectrum agents based on culture results	Reduces resistance, minimizes adverse effects	Requires rapid diagnostic testing
Biomarker-Guided Therapy	Use of procalcitonin or CRP to tailor duration	Avoids unnecessarily prolonged therapy	Not always available in resource-limited settings
Outpatient Parenteral Antimicrobial Therapy (OPAT)	Use of long-acting agents for outpatient management	Decreases hospital stay, improves patient convenience	Lipoglycopeptides are ideal for OPAT
AI-Driven Decision Support	Machine learning models analysing EHRs to predict the optimal duration	Enhances precision in antibiotic selection and duration	Requires integration into clinical workflows
Rapid Molecular Diagnostics	Faster pathogen identification and resistance profiling	Enables early de-escalation, preventing overtreatment	Adoption varies across healthcare settings

## Data Availability

Not applicable.
